# *SLC26A4*-linked CEVA haplotype correlates with phenotype in patients with enlargement of the vestibular aqueduct

**DOI:** 10.1186/s12881-019-0853-4

**Published:** 2019-07-02

**Authors:** Janet R. Chao, Parna Chattaraj, Tina Munjal, Keiji Honda, Kelly A. King, Christopher K. Zalewski, Wade W. Chien, Carmen C. Brewer, Andrew J. Griffith

**Affiliations:** 1 Otolaryngology Branch, National Institute on Deafness and Other Communication Disorders, National Institutes of Health, 35A Convent Drive, Room GF103, NIDCD/NIH, Bethesda, MD USA; 2 Division of Otolaryngology, Department of Surgery, Yale School of Medicine, New Haven, CT USA; 3 Neurotology Program, National Institute on Deafness and Other Communication Disorders, National Institutes of Health, Bethesda, MD USA

**Keywords:** Deafness, DFNB4, Haplotype, Hearing, Noncoding, Pendred syndrome, *SLC26A4*

## Abstract

**Background:**

Recessive mutations of coding regions and splice sites of the *SLC26A4* gene cause hearing loss with enlargement of the vestibular aqueduct (EVA). Some patients also have a thyroid iodination defect that can lead to multinodular goiter as part of Pendred syndrome. A haplotype of variants upstream of *SLC26A4*, called CEVA, acts as a pathogenic recessive allele in *trans* to mutations affecting the coding regions or splice sites of *SLC26A4*. Our first hypothesis is that CEVA, acting as a pathogenic recessive allele, is correlated with a less severe phenotype than mutations affecting the coding regions and splice sites of *SLC26A4*. Our second hypothesis is that CEVA acts as a modifier of the phenotype in patients with EVA caused by mutations affecting the coding regions or splice sites of both alleles of *SLC26A4* or EVA caused by other factors.

**Methods:**

This was a prospective cohort study of 114 individuals and 202 ears with EVA**.** To test our first hypothesis, we compared the thyroid and auditory phenotypes of subjects with mutations affecting coding regions of both alleles of *SLC26A4* with those of subjects carrying CEVA in *trans* to mutations affecting the coding regions. To test our second hypothesis, we compared the phenotypes associated with the presence versus absence of CEVA among subjects with no coding region mutations, as well as among subjects with mutations affecting coding regions of both alleles.

**Results:**

Subjects carrying CEVA in *trans* to a mutation of *SLC26A4* have a normal thyroid phenotype and less severe hearing loss in comparison to individuals with mutations affecting coding regions of both alleles of *SLC26A4*. In subjects with no mutant alleles of *SLC26A4*, hearing loss was more severe in subjects who carry the CEVA haplotype in comparison to non-carriers. There was no correlation of CEVA with the phenotype of subjects with mutations affecting coding regions of both alleles.

**Conclusions:**

CEVA, acting as a likely pathogenic recessive allele, is associated with a less severe phenotype than alleles with a mutation affecting the coding regions or splice sites of *SLC26A4*. CEVA may act as a genetic modifier in patients with EVA caused by other factors.

## Background

Enlarged vestibular aqueduct (EVA) is the most common temporal bone malformation detected in ears with sensorineural hearing loss [1]. Some individuals with EVA have recessive mutations of the coding regions and splice sites of the *SLC26A4* gene on chromosome 7q (OMIM 274600) [2]. Mutations of *SLC26A4* can cause bilateral EVA and a thyroid iodination defect that can lead to multinodular goiter as part of Pendred syndrome (PS) (OMIM 274600) [3]. The iodination defect can be detected with the perchlorate discharge test [4]. The same pathogenic variants of *SLC26A4* can also be associated with nonsyndromic EVA (NSEVA), also referred to as nonsyndromic recessive hearing loss DFNB4 (OMIM 600791) [2, 5].

Until recently, there was no known correlation of specific mutations or variants of *SLC26A4* with the presence or absence of the PS thyroid phenotype. Variants originally reported to be specifically associated with NSEVA/DFNB4 [6] are now thought to be coincidental hypomorphic variants [7]. However, it was recently reported that some specific mutations are correlated with hearing loss progression or severity in Korean populations [8, 9].

We have reported correlations of the number of mutant alleles of *SLC26A4* with the thyroid phenotype [10, 11], bi−/unilaterality of EVA [11], severity of hearing loss [12, 13], and recurrence probability of EVA in siblings of Caucasian EVA probands [14]. Pendred syndrome, as defined by an abnormal perchlorate discharge test result, is correlated with biallelic mutations of *SLC26A4* (M2) [11]. This correlation is less strong when PS is defined as the presence of multinodular goiter. M2 patients almost always have bilateral EVA, whereas unilateral EVA is almost always associated with one (M1) or zero (M0) mutant alleles of *SLC26A4* [11]. Severity of hearing loss is greater in M2 EVA ears than in M0 or M1 EVA ears [12, 13].

We recently reported an *SLC26A4*-linked haplotype, called Caucasian EVA (CEVA), comprised of 12 variants (10 single-nucleotide substitutions and two single-nucleotide deletions) upstream of *SLC26A4* [15]. The frequency of the CEVA haplotype among independent control cohorts, not ascertained for hearing loss, was 28 of 947 chromosomes among Europeans and 11 of 676 chromosomes among Admixed Americans [15]. CEVA was far less frequent among Africans (1 of 1314 chromosomes), East Asians (0 of 1008 chromosomes), and South Asians (1 of 971 chromosomes) [15]. The 12 variants span 613 kb and five other genes within a region of linkage disequilibrium that extends upstream from the first intron of *SLC26A4*. Some of the variants are intergenic and some are found within introns of the other genes which include *BCAP29*, *DUS4L*, *COG5*, *HBP1*, and *PRKAR2B*. None of these five genes are known to be associated with EVA or any phenotype consistent with EVA. We showed that a chromosome 7 with CEVA and no mutations of coding regions or splice sites of *SLC26A4* acts as a mutant allele with incomplete penetrance in *trans* to the allele with a mutation affecting the coding regions or splice sites in M1 patients [15]. The prevalence of CEVA was also elevated among Caucasian M0 EVA subjects, although it did not seem to be necessary or sufficient for the etiology of EVA in M0 subjects [15].

Based on these findings, we hypothesized that the *SLC26A4* allele with the CEVA haplotype is correlated with a less severe phenotype than mutations affecting the coding regions or splice sites of *SLC26A4*. The aim of our current study was to test this hypothesis by comparison of the phenotypes of M2 subjects without CEVA with those of M1 subjects carrying CEVA in *trans* to a mutation affecting the coding regions or splice sites. A second aim of our study was to test the hypothesis that CEVA acts as a genetic modifier of the phenotype caused by mutations affecting coding regions or splice sites of both alleles of *SLC26A4* (M2) or by factors other than *SLC26A4* mutations (M0).

## Methods

### Subjects

This study was approved by the Combined Neurosciences Institutional Review Board (IRB) of the National Institutes of Health (Bethesda, Maryland, USA). Written informed consent was obtained from all adult subjects and parents of minor subjects. Race and ethnicity were classified according to our IRB reporting designations. All Caucasians with EVA on at least one side were included in this study. We originally defined a vestibular aqueduct as enlarged if the diameter > 1.5 mm [16], but subsequently revised our criterion to > 1.0 mm at the midpoint of the course of the vestibular aqueduct [17]. Subjects were categorized as having either unilateral (U) or bilateral (B) EVA. We excluded subjects without original radiologic images to confirm the presence of EVA. *SLC26A4* genotypes and CEVA haplotypes have been previously reported [7, 11, 15, 18, 19]. We excluded three subjects whose haplotype was neither CEVA nor reference [15]. Two previously reported subjects [11] (1159 and 1171) could not be included due to absence of samples. Our cohort included 202 ears with EVA in 114 subjects: 88 subjects with bilateral EVA with 26 subjects with unilateral EVA (Table 1). Thirty-one subjects had homozygous or compound heterozygous pathogenic variants (M2), 14 subjects had one pathogenic variant (M1), and 69 subjects had no pathogenic variants affecting splice sites or coding regions of *SLC26A4* (M0).

**Table 1:** Study subject demographics

Number of Mutant Alleles of *SLC26A4*	*SLC26A4*-linked haplotype	Number of subjects (male/female)	Average age of subjects (years)	Number of ears (male/female)	Number of analyzed ears^a^ (male/female)	Average age of analyzed ears^a^ (years)
2	C/C	1 (0/1)	4.8	2 (0/2)	2 (0/2)	4.8
	C/R	3 (1/2)	10.7	6 (2/4)	6 (2/4)	10.7
	R/R	27 (11/16)	17.4	52 (21/31)	48 (20/28)	18.4
1	C/R^b^	11 (6/5)	7.8	20 (10/10)	20 (10/10)	7.5
	R/R	3 (2/1)	19.7	5 (3/2)	5 (3/2)	15.8
0	C/C	4 (2/2)	12.4	7 (4/3)	7 (4/3)	13.0
	C/R	6 (3/3)	12.4	10 (5/5)	6 (3/3)	10.1
	R/R	59 (22/37)	11.3	100 (39/61)	94 (34/60)	12.9
Total		114 (47/67)	12.6	202 (84/118)	188 (76/112)	13.6

*C* CEVA, *R* reference (most common haplotype)

^a^Ears with sufficient audiometric data for analysis

^b^ CEVA in *trans* to mutant allele of *SLC26A4*

### Thyroid phenotype

Two of 114 subjects were excluded from the thyroid phenotype analysis due to a lack of any testing or reports. Thyroid phenotype was categorized as non-syndromic (NS), Pendred syndrome (PS), or indeterminate (I). Ultrasonography was used to evaluate thyroid size and texture [10, 11]. Subjects with an elevated perchlorate discharge, regardless of thyroid texture or size, were categorized as PS [10, 11]. Subjects were categorized as NS if they had normal thyroid size, normal thyroid texture, and normal perchlorate discharge (≤15%). Subjects were categorized as I if they had any of the following: (1) no record of a perchlorate discharge test; (2) no record of a thyroid ultrasound test; (3) no record of thyroid serologic tests to rule out concurrent confounding thyroid phenotypes; (4) normal perchlorate discharge, normal thyroid texture, and increased thyroid size; or (5) normal perchlorate discharge and a multinodular, enlarged thyroid. One subject (2085) with normal thyroid texture and size but a borderline perchlorate discharge was categorized as I. Subjects with an I phenotype were excluded from further analysis.

### Auditory phenotype

Severity of hearing loss was classified using a four-frequency (0.5/1/2/4-kHz) pure-tone air-conduction threshold average (PTA) calculated from the most recent complete audiogram for each ear with EVA [12, 13]. When there was no response to an air-conducted stimulus, we added 5 dB to the maximum output of the audiometer (i.e. the “no response” value) and used that value as a proxy for analysis. Ears were excluded if there was not at least one complete pure-tone air-conduction audiogram for the ear with EVA prior to or without a cochlear implant [12, 13].

### Statistical analyses

Fisher’s exact test was used to investigate associations of sex, EVA laterality (unilateral vs. bilateral), and thyroid phenotype with *SLC26A4* genotype-haplotype combination. The following comparisons were made: (1) M2 vs. M1/CEVA (2) M2 vs. M2/CEVA (3) M0 vs. M0/CEVA. The Mann-Whitney test was used to compare pure-tone threshold average (PTA) for ears with different genotype-haplotype combinations. These analyses were performed using GraphPad Prism 7 for Mac OS X (GraphPad Software, La Jolla, CA). Stata (StataCorp LLC, College Station, Texas) was used to perform multivariate linear regression analysis to investigate associations of hearing loss in M2 R/R and M1 C/R EVA patients as a function of genotype status (M1 or M2), CEVA (reference or CEVA) haplotype status, age, sex, and EVA laterality. We performed the same analysis in M0 EVA patients but without genotype status as a variable since all patients were M0.

## Results

**Fig. 1:**
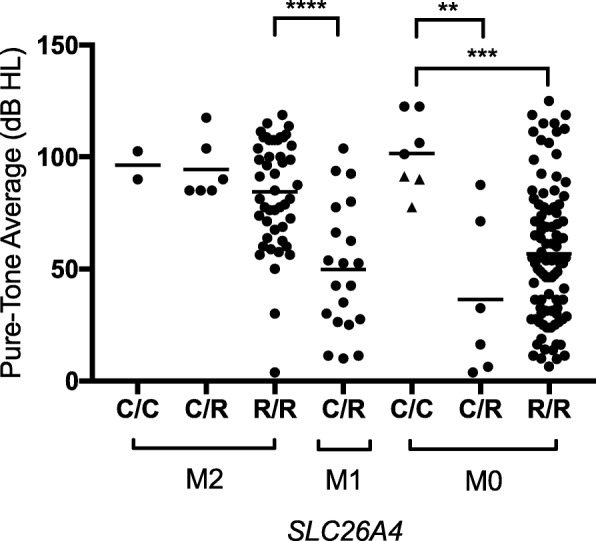
Four-frequency (0.5/1/2/4 KHz) pure-tone threshold averages for ears with enlargement of the vestibular aqueduct. Each data point represents one ear displayed according to *SLC26A4* genotype status (M0, M1 or M2) and haplotype status (C, CEVA; R, reference (most common haplotype)). The CEVA haplotype is *trans* to the *SLC26A4* mutation in the M1 group. **p ≤ 0.01, ***p ≤ 0.001, ****p ≤ 0.0001, Mann-Whitney Rank Test. M0 data points shown as a triangle (▲) correspond to CEVA homozygotes with one allele in *cis* with p.M775 T, a hypofunctional variant thought to be pathogenic only in *trans* with a mutation affecting the coding region or splice sites

To test the hypothesis that CEVA, acting as a recessive mutant allele, is correlated with a less severe phenotype than mutations affecting the coding regions or splice sites of *SLC26A4,* we compared the phenotypes of M2 subjects without CEVA to those of M1 subjects with CEVA in *trans* to an allele with the reference haplotype and a mutation affecting the coding regions or splice sites but no CEVA. Among subjects with a determinate thyroid phenotype (Pendred syndrome or nonsyndromic), 10 of 11 M2 subjects without the CEVA haplotype had a Pendred syndrome thyroid phenotype and six of six M1 subjects with CEVA in *trans* had a nonsyndromic phenotype (Table 2). This difference was significant (*p* = 0.0006; OR = ∞). Twenty-five (93%) of 27 M2 subjects without CEVA and nine (82%) of 11 M1 subjects with CEVA in *trans* had bilateral EVA. This difference was not significant (*p* = 0.564). The median pure-tone average (86.3 dB HL; *n* = 48 ears with sufficient audiometric data) in the M2 without CEVA group was significantly different (*p* < 0.0001; 95% CI of difference = − 52.5 to − 21.3) from the median pure-tone average (47.5 dB HL; *n* = 20 ears with sufficient audiometric data) in the M1 with CEVA group (Fig. 1). Linear regression analysis determined that among M2 R/R and M1 C/R patients, there is a significant negative correlation between hearing loss and CEVA (patients with CEVA have less severe hearing loss), adjusting for age, sex, and laterality (*p* < 0.001; R^2^ = 3.94). These results indicate that, as a recessive Mendelian allele in *trans* to an allele with a mutation of the coding regions or splice sites of *SLC26A4*, an allele with CEVA but no coding region or splice site mutations is associated with a normal thyroid phenotype and less severe hearing loss in comparison to alleles with a mutation of the coding regions or splice sites and the reference haplotype.

**Table 2:** Thyroid phenotypes of study subjects

		Number of subjects		
Number of Mutant Alleles of *SLC26A4*	*SLC26A4*-linked haplotype	Non-syndromic	Pendred syndrome	Indeterminate
2	C/C	0	0	1
	C/R	0	1	2
	R/R	1	10	16
1	C/R	6	0	5
	R/R	1	0	2
0	C/C	1	0	3
	C/R	0	0	6
	R/R	29	0	27
Total		38	11	62

*NS* non-syndromic, *PS* Pendred syndrome, *I* indeterminate, *C* CEVA, *R* reference

To test the hypothesis that CEVA acts as a modifier of the phenotype of patients with EVA caused by factors other than *SLC26A4* mutations, we compared the phenotypes of M0 subjects without CEVA with the phenotypes of M0 subjects heterozygous or homozygous for the CEVA haplotype (homozygous *SLC26A4* alleles with the CEVA haplotype do not act as pathogenic recessive alleles in M0 subjects [15]). Among M0 subjects with a determinate thyroid phenotype, 31 (100%) of 31 subjects had a nonsyndromic phenotype irrespective of CEVA haplotype status. Eighteen (31%) of 59 M0 subjects without CEVA and three (30%) of 10 M0 subjects with CEVA had unilateral EVA. This difference was not significant (*p* > 0.999). The median pure-tone average (54.4 dB HL; *n* = 94 ears with sufficient audiometric data) in the M0 without CEVA group was significantly different (*p* = 0.0002; 95% CI of difference = − 1.25 to 51.3) than the median pure-tone average (101.3 dB HL; *n* = 7 ears with sufficient audiometric data) in the M0 with homozygous CEVA group (Fig. 1). The latter subjects also had more severe hearing loss (*p* = 0.0023; 95% CI of difference = 18.8 to 102.5) than M0 subjects heterozygous for CEVA (24.4 dB HL; *n* = 6 ears with sufficient audiometric data) (Fig. 1). Linear regression analysis confirmed this correlation after adjusting for age, sex, and EVA laterality (*p* = 0.003; R^2^ = 16.8). These results indicate that CEVA modifies the severity of hearing loss in M0 EVA ears.

We did not observe any significant differences between hearing loss severity and number of *SLC26A4* alleles with the CEVA haplotype within the cohort of M2 subjects (*p* = 0.3346). These results do not support the hypothesis that CEVA modifies the severity of hearing loss caused by mutations affecting the coding regions or splice sites of both alleles of *SLC26A4*.

## Discussion

We recently reported a haplotype of 12 variants upstream of *SLC26A4*, termed CEVA, that acts as a pathogenic recessive allele in *trans* to an allele with a mutation affecting the coding regions or splice sites of *SLC26A4* in M1 EVA patients [15]. When American College of Medical Genetics and Genomics criteria are applied to CEVA, there are strong (PS4), moderate (PM5) and supporting (PP1) lines of evidence for its pathogenicity [15], resulting in overall classification as “likely pathogenic.” [20].

In contrast to individuals with mutations affecting coding regions or splice sites of both alleles of *SLC26A4*, individuals carrying an *SLC26A4* allele with the CEVA haplotype in *trans* to a mutation of *SLC26A4* have a normal thyroid phenotype (i.e. nonsyndromic EVA or DFNB4) and tend to have less severe hearing loss. Although our previous comparisons of phenotypes of M2 subjects with those of M1 subjects revealed that the M1 phenotypes were less severe than the M2 phenotypes, some of the M1 subjects had the reference haplotype in *trans* to the mutant allele, and some of the M2 subjects had CEVA in *cis* with one or both mutant alleles. Our current result and conclusion are novel because we performed a comparison that was not confounded by inclusion of M1/reference or M2/CEVA subjects.

Our analysis of M0 subjects suggests that CEVA, acting as a genetic modifier, increases the severity of hearing loss associated with EVA caused by factors other than mutant alleles of *SLC26A4*. Alternatively, our observation may result from ascertainment bias or other unknown factors among the M0 subjects. In contrast, we did not observe any correlations between hearing loss severity and number of *SLC26A4* alleles with the CEVA haplotype within the cohort of M2 subjects. However, the power of these analyses was limited by the number of M2 subjects with CEVA (four). We did not perform this analysis in M1 subjects since CEVA is etiologic in those subjects and cannot also be considered a modifier. Furthermore, we cannot be certain of the etiology of EVA in M1 subjects without CEVA. We could therefore not test the hypothesis that CEVA acts as a genetic modifier in M1 subjects.

It is possible there are pathogenic variants in regions or genes unlinked to *SLC26A4* that cause hearing loss and nonsyndromic EVA or modify the severity of hearing loss in ears with EVA. However, the co-segregation of EVA with *SLC26A4*-linked markers in M1 families, and the near-zero probability of EVA in the siblings of M0 EVA probands, indicate that such variants would be extremely rare Mendelian causes of nonsyndromic EVA [14]. Our results do not address whether variants in unlinked regions and genes modify the hearing loss phenotype in ears with EVA.

We previously recommended inclusion of testing for CEVA along with analysis of *SLC26A4* exons and splice sites for individuals with hearing loss and EVA because the detection of CEVA provides a definitive diagnosis for individuals with a mutation affecting the *trans* allele of *SLC26A4* [15]. Our current study directly demonstrates that individuals with this *SLC26A4* genotype-haplotype result are unlikely to develop the thyroid abnormality and increased severity of hearing loss associated with Pendred syndrome and mutations affecting the splice sites or coding regions of both alleles of *SLC26A4*. Finally, this study indicates that CEVA may act as a genetic modifier to increase the severity of hearing loss in ears with EVA caused by factors other than mutations affecting the coding regions or splice sites of *SLC26A4*.

## Conclusions

CEVA, acting as a likely pathogenic recessive allele of *SLC26A4*, is associated with less severe auditory and thyroid phenotypes than alleles with a mutation affecting the coding regions or splice sites of *SLC26A4*. CEVA may act as a genetic modifier in patients with EVA caused by other factors.

## Data Availability

The data that support the findings of this study are available from the corresponding author upon reasonable request.

## Abbreviations

CEVA: *SLC26A4*-linked haplotype associated with enlargement of the vestibular aqueduct
EVA: Enlargement of the vestibular aqueduct
I: Indeterminate thyroid phenotype
M0: Harboring no mutations affecting coding regions or splice sites of *SLC26A4*
M1: Harboring a mutation affecting coding regions or splice sites of one allele of *SLC26A4*
M2: Harboring mutations affecting coding regions or splice sites of both alleles *SLC26A4*
NSEVA: Nonsyndromic enlargement of the vestibular aqueduct
PS: Pendred syndrome
PTA: Pure-tone (hearing) threshold average
